# The forgotten value of the clinical examination to individualize and guide fluid resuscitation in patients with sepsis

**DOI:** 10.1186/s13054-017-1898-4

**Published:** 2017-12-19

**Authors:** Gentle Sunder Shrestha, Martin Dünser, Mervyn Mer

**Affiliations:** 1 Department of Anaesthesiology, Critical Care Unit, Tribhuvan University Teaching Hospital, Maharajgunj, Kathmandu, Nepal; 2 Clinic of Anesthesiology and Intensive Care, Kepler University Clinic, Johannes Kepler University Linz, Altenbergerstrasse 69, 4040 Linz, Austria; 3 Divisions of Critical Care and Pulmonology, Department of Medicine, Charlotte Maxeke Johannesburg Academic Hospital and Faculty of Health Sciences University of the Witwatersrand, Johannesburg, South Africa

Andrews et al. reported that protocol-driven fluid resuscitation was associated with enhanced lactate clearance but offset by more respiratory distress and substantially higher mortality in patients with sepsis in the sub-Saharan African setting with limited access to mechanical ventilators [1]. Lack of access to oxygen and ventilation facilities is a frequent and widespread challenge in hospitals in low- and middle-income countries (LMICs), where approximately three-quarters of the world’s population reside. This needs to be considered while using contemporary international guidelines on the management of sepsis, which are largely based on scientific evidence originating from high-income settings [2, 3].

Although the trial results presented by Dr. Andrews may ostensibly appear negative, we do not necessarily concur. When focusing on study patients referred to the usual care group, it is evident that their in-hospital mortality was surprisingly low despite concomitant severe co-morbidities, including immunosuppression (90% HIV infected), malnutrition, anemia, and sepsis with arterial hypotension, in a setting where critical care facilities were essentially unavailable. Indeed, the mortality predicted by a Simplified Acute Physiology Score III count of 57 is not significantly lower than the one reported (29 vs. 33%; odds ratio, 0.83; 95% confidence interval, 0.44–1.57; *p* = 0.65) [4]. Consequently, we feel it would be incorrect to conclude that the risks of intravenous fluid resuscitation generally outweigh benefits in sepsis patients in settings where critical care and mechanical ventilators are not readily accessible. Evaluation of individual risk factors in conjunction with careful clinical examination performed by the treating clinician directed fluid resuscitation in about half of the patients, with a median amount of 2 L in the first 6 h being administered to the usual care group. This resulted in comparable increases in arterial blood pressure and less respiratory distress than in the study group, albeit with a slower lactate clearance.

In summary, we believe that these trial findings admirably highlight the relevance and benefits of the clinical examination and acumen over a non-individualized protocol to guide early fluid resuscitation in patients with sepsis in LMICs. Although extrapolation to other settings appears complex, these results should remind clinicians about the fundamental, essential and vital role of the clinical examination, a technique which is often underappreciated in resource-rich settings [5] but which still represents an indispensible tool to guide resuscitation in sepsis patients in LMICs [3]. In an era of ever improving and advancing technology and protocolized care, sound clinical skills and acumen should never be forgotten—it costs nothing, should be readily available, and saves lives!

## Abbreviations

LMICs: Low- and middle-income countries.
